# Editorial: Genomics and disease resistance in wheat and maize

**DOI:** 10.3389/fpls.2022.1064948

**Published:** 2022-11-15

**Authors:** Cheng Liu, Handong Su, Shun Sakuma, Mingliang Xu, James A. Birchler, Fangpu Han

**Affiliations:** ^1^ Crop Research Institute, Shandong Academy of Agricultural Sciences, Jinan, China; ^2^ National Key Laboratory of Crop Genetic Improvement, Hubei Hongshan Laboratory, Shenzhen Institute of Nutrition and Health, Huazhong Agricultural University, Wuhan, China; ^3^ Tottori University, Tottori, Japan; ^4^ China Agricultural University, Beijing, China; ^5^ University of Missouri, Columbia, SC, United States; ^6^ Institute of Genetics and Developmental Biology, Chinese Academy of Sciences, Beijing, China

**Keywords:** disease resistance, gene mapping, distant hybridization, gene structure, wheat, maize

## Introduction

Due to the extreme climatic events and inappropriate cropping patterns, numerous diseases are becoming more and more serious for wheat and maize in recent years, which causes yield losses and affects food security worldwide (Krupinsky et al., 2002; Parikka et al., 2012). Fusarium head blight, powdery mildew, and rusts are the most serious diseases of wheat (*Triticum aestivum* L.) (Liu et al., 2020). Stalk rot, head smut, southern corn rust, and ear rot are among the most serious diseases that can substantially reduce maize yield and impact global markets (Zhu et al., 2021). New types or variants of phytopathogens overcome past sources of resistance with the ever-shrinking genetic diversity of crop varieties (Li et al., 2009; Liu et al., 2020). With the rapid advance in genomics tools, genetic and genomic resources are now being the key approach for basic research and breeding for the crop disease resistance community (Feng et al., 2018; Liu et al., 2020). Hence, there is an urgency to explore novel disease resistance genes and their mechanisms of action in wheat and maize. In this topic, recent advances in genomics and disease resistance or stress tolerance studies for wheat and maize are presented in 15 publications, contributed by 131 authors.

## Genome assembly and gene structure

Genomic assembly of the pathogen is helpful to understand its pathogenesis. Ma et al. sequenced and assembled the whole genome of *Didymella glomerata*, a new fungal pathogen causing Didymella leaf blight (DLB) in maize. They identified three maize germplasms conferring resistance to DLB, and revealed potential mechanism underlying DLB resistance. By subjecting wheat to ethyl methane sulfonate treatment, He et al. created 113 mutations in the coding region of the *Pm21* gene that encodes a broad-spectrum resistance to powdery mildew, and revealed the key functional sites for resistance and structural distribution characteristics. Sun et al. analyzed the expression pattern of the type-A response regulatory gene family under different stresses in wheat.

## Disease resistance gene mapping or mining

It is very important to locate the disease resistance genes and explore their alleles for studying their genetic evolution and evaluating their breeding value in crops (Fu et al., 2012). Sun et al. mapped a new powdery mildew resistance gene *Pm_SN15218_* on wheat chromosome 2AL from the breeding line SN15218, which is distinct from the known resistance gene *Pm4b*. Mu et al. identified a recessive powdery mildew resistance gene *pmYN99102* on wheat chromosome 2BL *via* bulked segregant exome capture sequencing. The gene can be traced when it is integrated into those susceptible cultivars. Yu et al. mined the alleles of wheat powdery mildew resistance gene *Pm2*, providing valuable information for the utilization of *Pm2* alleles in wheat breeding. Tong et al. verified the great breeding value of the maize *ZmCCT* haplotype *H5*, which synchronously modulates the yield potential, stalk-rot resistance, and drought tolerance. Lv et al. proposed that different adaptive patterns played important roles under complex drought tolerance based on integrated transcriptome and metabolome profiling.

## Mapping quantitative disease resistance loci

Mapping QDR locus is a critical for cloning and utilizing the resistance gene resources in crops. Bai et al. mapped three new QDR loci from wheat cultivar “Pascal” with resistance to stripe rust at the adult plant stage using a recombinant inbred line population. Zhang et al. found four possible new FHB resistance loci in hard winter wheat germplasm using a multi-locus genome-wide association study. Xia et al. fine mapped a Fusarium ear rot resistance gene in maize by QTL mapping and RNA sequencing. Zhu et al. performed a high-resolution mapping of a *Helminthosporium turcium resistance 3*-like locus against north corn leaf blight.

## Creation of disease resistant distant hybrid germplasm

Distant hybrid material with disease resistance is an important bridge for crop breeding (Liu et al., 2011; Liu et al., 2020). Tian et al. developed and characterized the *Triticum aestivum-Aegilops longissima* recombinants using the CS *ph1b* mutant as an inducing tool, which harbors a novel powdery mildew resistance gene *Pm6Sl*. Duan et al. narrowed down the candidate region of stripe rust resistance gene *Yr83* using newly developed wheat-rye chromosome translocations. These small-segment translocation materials are promising for the improvement of wheat cultivars. Ren et al. developed new wheat-rye 6R, 6RS, and 6RL addition lines, and identified novel resistance genes to stripe rust and powdery mildew.

## Author contributions

CL compiled the contributions from all authors. All authors approved the final version of the manuscript and approved it for publication.

## Funding

This work was supported financially supported by Taishan Scholars Project (tsqn201812123) and National Natural Science Foundation of China (31971847).

## Acknowledgments

We greatly appreciate the contributions from all the authors and reviewers as well as the support of the editorial office of Frontiers in Plant Science.

## Conflict of interest

The authors declare that the research was conducted in the absence of any commercial or financial relationships that could be construed as a potential conflict of interest.

## Publisher’s note

All claims expressed in this article are solely those of the authors and do not necessarily represent those of their affiliated organizations, or those of the publisher, the editors and the reviewers. Any product that may be evaluated in this article, or claim that may be made by its manufacturer, is not guaranteed or endorsed by the publisher.
